# Empowering women in sustainable agriculture

**DOI:** 10.1038/s41598-024-57933-y

**Published:** 2024-03-26

**Authors:** Imre Fertő, Štefan Bojnec

**Affiliations:** 1 HUN-REN Centre for Economic and Regional Studies, Tóth K. u. 4, 1112 Budapest, Hungary; 2 Corvinus University of Budapest, Fővám tér 8, 1097 Budapest, Hungary; 3 Czech University of Life Sciences, Kamycka 129, Prague, Czech Republic; 4 University of Primorska, Izolska vrata 2, 6000 Koper, Slovenia

**Keywords:** Climate change, Gender, Agri-environment-climate measures, Subsidies, Decomposition models, European Union, Environmental sciences, Environmental social sciences

## Abstract

The agricultural and rural development policy seeks to facilitate the transition towards environmentally sustainable and climate-neutral agricultural practices, with a focus on human capital, knowledge, and innovation. Gender equality can play a significant role in promoting environmentally sustainable practices in the agricultural sector, particularly through the adoption and implementation of agri-environment-climate schemes (AECS) in the context of farm, agricultural, and rural development. We examine the presence of gender bias in the adoption intensity of AECS by utilising farm-level data from Slovenia. We find that women on Slovenian farms engage in the adoption of AECS and receive subsidies, despite the presence of a gender gap in various agricultural factor endowment variables that typically favour men. The results of this study provide evidence in favour of promoting greater involvement and empowerment of women in the fields of green technology applications and green entrepreneurship, particularly with AECS practices.

## Introduction

The application of green agricultural technologies and farming practices can play an important role in the mitigation of climate change with impacts on the rural economy, environment and society^1–3^. Tackling climate change action with the adoption of sustainability of farming practices to accelerate sustainable development gains importance in interdisciplinary research, policy and society responses^4–6^.

The policy response to climate change in agriculture is through measures of agricultural policy^7,8^. In the European Union (EU) countries, the agri-environment-climate scheme (AECS) measures are introduced within the Common Agricultural Policy (CAP)^9,10^. While there are studies to investigate the impacts of AECS such as on farmland biodiversity^11^, farm performance^12^, farm employment^13^, groundwater quality^14^, and adoption of the AECS in EU agriculture and policy modelling of economic, sustainability and development effects^15–18^, there is no study to investigate drivers of adoption and intensity of AECS concerning the gender^19,20^. This gap in the literature has motivated our research^21^.

Gender equality is one of the objectives for sustainable rural development relevant to policy and governance with wider implications for the rural economy, green entrepreneurship and society^19,20,22^. We shed light on drivers of AECS adoption and its intensity in the EU country focusing on the role of women-headed farms in green farming practices.

Gender equality and rural women’s empowerment can drive farm and rural entrepreneurship in green transitions from peasants to more entrepreneurial and resilient farming and rural society. Women-led green entrepreneurship in farming and the rural economy can develop in different economic activities^23^. Women’s participation in green farming and rural entrepreneurship is a relatively new phenomenon. The green on-farming activities can be measured in different ways, often with the voluntary participation of farms in AECS^15,24^.

Agricultural policy plays a key role in shaping the pro-environment-climate behaviour of farmers, which includes such basic mechanisms as regulations and economic instruments which pay farmers directly for adopting environmentally friendly practices. Recognition of the motives and factors encouraging farmers to participate in AECS is particularly important in the context of voluntary adoption of conservation practices in most of these programmes^7,16^. The willingness of farmers to participate in such programmes is a necessary condition, although it does not guarantee success in achieving the assumed resilience and sustainability goals.

A body of literature on the determinants of participation in AECS in different countries has developed^17,25^, but results from various countries remain ambiguous. This indicates that many conditions are not only country but local-specific and require more detailed recognition in different geographical or spatial contexts. More recently, they^26^ emphasize the role of non-cognitive skills, namely self-efficacy, and locus of control, in farmers’ uptake of mitigation measures. However, there is no research on gender-driven participation in the AECS in the Central and Eastern European (CEE) countries.

The main objective of this paper is to analyse the differences in the AECS adoption and intensity between male- and female-headed Slovenian FADN farmers. Slovenia belongs to the group of the CEE countries, which are the members of the EU and its CAP. Therefore, the results and findings can be relevant and important also for some other EU member states. Unlike any previous studies, we employ the Blinder–Oaxaca (B–O) decomposition panel model econometric approach^27,28^. Finally, the study is relevant for science, policy and practice on the gender-driven participation and intensity in the AECS that can contribute to farm and agricultural sustainability.

## Methodology

### Blinder-Oaxaca decomposition

The Blinder-Oaxaca (B–O) decomposition model has been predominantly used in labour economics literature to study gaps in wages and employment^27,28^, which has later been applied in agricultural productivity gap studies^29,30^. B–O decomposition is not path-dependent and quantifies the relative contribution of factors to the gap. We employ a threefold decomposition, namely, the AECS intensity gap is divided into three parts. First, the endowment effect reflects the mean increase in women’s AECS intensity if they had the same characteristics as men. Second, the coefficient effect quantifies the change in women’s AECS intensity when applying the men’s coefficients to the women’s characteristics. Third, the interaction effect measures the simultaneous effect of differences in endowments and coefficients. However, the AECS subsidies are observed only for farmers who are participating in the AECS programme, and this might be a selective group. Thus, we estimate the B-O model with the selectivity bias^31–33^.

### Data

We use the Slovenian Farm Accountancy Data Network (FADN) panel datasets between 2014 and 2021. The FADN is used as an informative source to monitor farms' income and business activities in EU countries and to understand the impact of the CAP measures. FADN provides farm-level data based on national surveys for agricultural holdings above the size threshold that can be considered commercial^34^. The farm-level data are provided according to regional farm location, the economic size of the farm, and its type of farming.

As a dependent variable in our regression models, we use three farm-level outcome variables linked to AECS subsidy: AECS subsidy, AECS subsidy/total CAP subsidy, and AECS subsidy/total utilized agricultural land. Explanatory variables at the farm-level are human capital variables, farm input variables (land, labour, and total assets), and total CAP subsidy. The type of farming activity is used to control for fixed effects in the regression models.

## Results

### Outcome variables for AECS adoption and AECS intensity

The descriptive statistics are presented for the four outcome variables linked to AECS subsidy at the farm-level separately for female- and male-headed farms: AECS adoption by the number of farms (in %), AECS subsidies (in euro), AECS subsidies/total CAP subsidies (in %), and AECS subsidies/total utilized agricultural land (in euro) (Table 1).

**Table 1:** Mean of variables.

	Male	Female	Total	Kruskal–Wallis test
AECS adoption (%)	63.3	67.2	64.1	0.0083
AECS subsidy (euro)	2580.10	1858.33	2435.68	0.6845
AECS/total CAP subsidy (%)	17.35	18.81	17.64	0.0039
AECS/total land (euro)	143.93	157.59	146.67	0.0031
Age (year)	43.84	46.18	44.31	0.0001
Education (1–3)	1.71	1.59	1.69	0.0001
Total labor (AWU)	1.51	1.49	1.51	0.4487
Unpaid labor (AWU)	1.40	1.40	1.40	0.4777
Total land (ha)	15.61	11.37	14.76	0.0001
Rented land (ha)	6.59	3.30	5.93	0.0001
Total output (euro)	47,732.03	32,052.69	44,594.78	0.0001
Total fixed asset (euro)	305,246.37	228,648.51	289,920.05	0.0001
Total subsidy (euro)	11,067.84	8101.49	10,474.31	0.0001
Fieldcrops	0.135	0.128	0.134	0.4939
Horticulture	0.017	0.008	0.015	0.0211
Wine	0.044	0.034	0.042	0.0856
Other permanent crops	0.070	0.066	0.069	0.6233
Milk	0.182	0.134	0.172	0.0001
Other grazing livestock	0.366	0.387	0.370	0.1646
Granivores	0.023	0.037	0.026	0.0048
Mixed	0.163	0.207	0.172	0.0001

The AECS measures are adopted by 64.1% of the Slovenian FADN farms, 67.2% by female-headed farms and 63.3% by male-headed farms. The voluntarily implemented AECS measures and the related AECS subsidies are constituent parts of CAP measures and subsidies. The share of AECS subsidies in total CAP subsidies is 17.6%, 18.8% for female-headed farms and 17.4% for male-headed farms. Other CAP subsidies for farms are still much more important than AECS subsidies for voluntarily implemented agri-environmental-climate measures.

Except for the AECS subsidy, female-headed farms received a higher AECS subsidy per hectare of agricultural land use (157.59 euro) than male-headed farms (143.93 euro). Female-headed farms experienced higher AECS adoption and higher intensity of AECS subsidies than male-headed farms.

The Kruskal–Wallis test confirms that except for AECS subsidy, the higher AECS adoption and intensity values for female-headed farms than for male-headed farms are statistically significant. Therefore, female-headed farms have more AECS adoption and have greater AECS intensity, as they received more AECS subsidy per hectare of agricultural land use and had a greater share of AECS subsidies in total CAP subsidies than men-headed farms. These findings are consistent with previous research^19^ arguing that farms run and operated by farm women are more agri-environmentally oriented than other farms.

### Explanatory variables

The descriptive statistics are presented for explanatory variables for human capital variables (gender, age, and education), farm input variables (land, labour, and total fixed assets), total output, and total CAP subsidies,—excluding investments. Farm size measured by total utilized agricultural land is 14.8 ha, 11.4 for females, and 15.6 ha for male-headed farms. There is observed a significant shift from traditional peasant farming to entrepreneurial and commercial farming. Namely, the percentage of the rented land is 27.8%, with variation from no rented land to completely or 100% rented land.

On average, farms employed 1.5 annual working units (AWU) of labour both on female- and male-headed farms mostly as unpaid family labour (1.4 AWU). These results and findings clearly confirmed the family-based nature of labour on the Slovenian farms.

Female-headed farms are significantly smaller than male-headed farms for total output, total fixed assets, and received total CAP subsidies per year. On average farms received 10,474.31 euros of total CAP subsidies per year, 8101.49 euros female-headed farms and 11,067.84 euros male-headed farms.

Gender inequality is also evident in Slovenian FADN farms, similar to other countries in the CEE region. The FADN sample is dominated by men with women representing only 20% of the sample. The average age is 44.3 years, 46.2 years for women and 43.8 years for men. Training is the medium magnitude on the scale between 0 and 3: 1.69 for all farms, 1.59 for females and 1.71 for male-headed farms.

According to the distribution of farms, female-headed farms are significantly more oriented in granivores and mixed farming, while male-headed farms in horticulture and dairy. Differences for other types of farming are not significant.

### Econometric results

The econometric results are presented for three outcome variables estimated by the B–O decomposition selection models: AECS adoption, AECS/total utilized agricultural land, and share of AECS in total CAP subsidies. We use these three dependent model variables to conduct a robustness check for both aggregate decomposition (Table 2) and detailed decomposition (Table 3).

**Table 2:** Results from Blinder–Oaxaca aggregate decomposition.

	AECS	AECS/total CAP subsidy	AECS/total land
Male	2580.103***	17.351***	143.933***
Female	5493.554***	45.798***	522.714***
Difference	− 2.9e+03***	− 28.448***	− 378.781***
Aggregate decomposition			
Endowments	739.527***	1.016	− 4.005
Coefficients	− 3.3e03***	− 28.125***	− 372.041***
Interaction	− 336.111**	− 1.339	− 2.735

*p < 0.1; ** p < 0.05; *** p < 0.01.

**Table 3:** Results from Blinder–Oaxaca detailed decomposition.

	AECS	AECS/total CAP subsidy	AECS/total land
Endowments’ effect			
Age	− 36.868	− 0.295	− 2.200
Education	− 31.946	− 0.151	− 2.949
Total labour	1.030	0.001	0.377
Unpaid labour	8.186	0.050	0.860
Total land	331.043	− 0.806	− 55.531**
Rented land	168.901	2.071	32.783
Total output	115.249	0.952	2.402
Total fixed asset	297.052**	1.886**	33.544**
Total subsidy	34.873	− 1.627**	− 3.987
Fieldcrops	− 9.960	− 0.107	− 0.608
Horticulture	0.719	0.006	0.191
Wine	17.172	0.223	2.792
Other permanent crops	− 18.349	− 0.209	− 2.881
Milk	− 115.947**	− 0.805**	− 6.453
Other grazing livestock	6.973	0.039	0.623
Granivores	− 28.601	− 0.212	− 2.967
Coefficients’ effect			
Age	− 432.970	− 5.389	− 44.331
Education	642.138	5.613*	93.293*
Total labour	− 392.120	− 0.221	− 24.378
Unpaid labour	311.034	4.232	85.763
Total land	1118.903	4.862	141.379*
Rented land	− 594.834**	− 2.440	− 35.087
Total output	− 313.961	− 2.554*	− 11.240
Total fixed asset	− 1.4e+03***	− 6.024**	− 95.598**
Total subsidy	2981.945***	12.088***	97.822*
Fieldcrops	− 114.287	− 1.121**	− 7.138
Horticulture	− 6.708	− 0.040	− 0.434
Wine	19.384	− 0.040	− 0.315
Other permanent crops	− 41.255	− 0.196	− 4.063
Milk	− 139.942**	− 0.115	− 0.636
Other grazing livestock	− 360.221**	− 1.376	− 12.015
Granivores	− 16.280	− 0.527**	− 6.929*
Interaction effect			
Age	15.954	0.202	1.638
Education	30.760	0.272	4.463
Total labour	− 6.987	− 0.004	− 0.437
Unpaid labour	− 2.754	− 0.034	− 0.751
Total land	331.005	1.450	41.891*
Rented land	− 498.279**	− 2.053	− 29.416
Total output	− 173.495	− 1.417*	− 6.216
Total fixed asset	− 503.118***	− 2.202**	− 34.813**
Total subsidy	524.193***	2.156***	17.230
Fieldcrops	11.544	0.111	0.719
Horticulture	− 4.568	− 0.027	− 0.296
Wine	7.695	− 0.016	− 0.125
Other permanent crops	7.030	0.035	0.691
Milk	− 110.231**	− 0.091	− 0.502
Other grazing livestock	29.983	0.112	0.996
Granivores	5.157	0.166	2.193

*p < 0.1; ** p < 0.05; *** p < 0.01.

While the gender gap in most studied agricultural factor endowment variables is in favour of men, women do make the difference in the adoption of ACES measures and received AECS subsidies on Slovenian farms. The B–O decomposition confirmed the gender gap in the received AECS subsidies (Table 2). The robustness tests confirmed that women received more AECS subsidies/total utilized agricultural land. Women received also the greater share of AECS subsidies in total CAP subsidies.

The B-O aggregate decomposition analysis shows that endowment effects play an important role in the received AECS subsidies, while the coefficients effect is dominant for the normalised outcome indicators: AECS subsidies/total CAP subsidies and AECS subsidies/total utilized agricultural land, respectively.

The B–O detailed decomposition confirmed that the increase in gender gap between women- and men-headed farms in received AECS subsidies is driven by total fixed assets and deteriorated by the dairy type of farming within the endowments effect. The change in women’s intensity within the coefficients effect is driven by total CAP subsidies and deteriorated by rented agricultural land and total fixed assets, as well as by dairy, other grazing, and granivore types of farming. Within the simultaneous interaction effect, the gender gap is driven by total CAP subsidies and deteriorated by rented agricultural land, total fixed assets and dairy type of farming.

Unlike our expectations, age and education with training are less important for receiving AECS subsidies. This finding can be related to the persistence of existing farming practices that can be less focused on the implementation of AECS measures.

The robustness tests confirmed that women’s increase in intensity of AECS subsidies per total CAP subsidies is due to total fixed assets, which is deteriorated by total CAP subsidies and dairy type of farming within the endowments effect. The change in women’s intensity within the coefficients effect is driven by total CAP subsidies and education and deteriorated by total output and total fixed assets as well as by field crops and granivores type of farming. Within the simultaneous interaction effect, the gender gap in AECS subsidies per total CAP subsidies is explained by total CAP subsidies and deteriorated by total output and total fixed assets.

The increase in women’s intensity vis-à-vis men in AECS subsidies per total utilized agricultural land is explained by total fixed assets, which is deteriorated by total utilized agricultural land. Within the coefficients effect, the change in women’s intensity in AECS subsidies per total utilized agricultural land is explained by total CAP subsidies, total utilised agricultural land and education, but deteriorated by total fixed assets and granivores type of farming. Within the simultaneous interaction effect, women vis-à-vis men intensity in AECS subsidies per total utilized agricultural land is driven by cultivation of total utilized agricultural land and deteriorated by total fixed assets.

The size of farms in terms of cultivation of total agricultural land and rented utilized agricultural land do matter for women’s increase/change in AECS intensity. The farm size reduces in women’s intensity gap in AECS subsidies per total utilized agricultural land intensity but increases the change in women’s intensity within the coefficients effect. Interestingly, a greater share of rented land reduces the change in women’s intensity in received AECS subsidies in the coefficients effect.

Age and both total labour and unpaid labour are insignificantly associated with AECS subsidies, AECS subsidies per total utilized agricultural land, and the share of AECS subsidies in total CAP subsidies. This finding is inconsistent with the previous research^13^ on green job creation in agriculture and in rural areas.

As the most striking finding, more capital-intensive farms with more total fixed assets and received more total CAP subsidies are significantly associated with received AECS subsidies, AECS subsidies per total utilized agricultural land, and the share of AECS subsidies in total CAP subsidies. This finding can be related to technological adjustments towards green AECS practices^35^. These results and findings can be important for the monitoring of CAP policies and the implementation of practices with raising awareness on the importance of green farming activities^36^.

## Discussion

Our results clearly confirmed that farms led by women exhibit a higher degree of environmental friendliness compared to farms led by men, both in terms of adoption and intensity of AECS measures. Therefore, it is important to prioritise the mitigation of gender disparities in farm leadership roles to promote climate-resilient development^37^. Additionally, it is crucial to emphasise the significance of educating and training rural women to empower them in pursue green entrepreneurship.

Furthermore, the adoption of AECS measures on farms should be encouraged. To implement these changes, technological advancements would need to be made, necessitating investments to enhance the overall infrastructure and fixed asset base within agricultural operations. The implementation of farm structural changes and the adoption of environmentally-friendly farming technologies, activities, and practices can be facilitated through the utilisation of existing CAP subsidies.

Enhanced gender equality within the agricultural sector in the CEE countries has the potential to yield numerous benefits. In addition to promoting environmentally friendly farming techniques in the cultivation of agricultural land, it has the potential to generate positive externalities that have yet to be thoroughly examined. These include advancements in demographic structures, mitigating depopulation, and fostering economic and social sustainability within farms and rural areas.

## Conclusions

Green agricultural technologies can mitigate climate change in the benefit of rural economies, environments, and societies. Sustainable farming practices are essential to interdisciplinary research, policymaking, and social action for mitigating climate change. To address climate change, the EU has implemented the AECS as part of the CAP, but gender dynamics in AECS adoption are poorly understood. The research aims to bridge this gap and offers insights into the role of women-headed farms in green farming practices within the context of the EU, specifically focusing on Slovenia.

The policy implications of this study are complex. The research emphasises gender equality as a key goal for sustainable farm and rural development. Rural women's empowerment may stimulate farm and rural entrepreneurship and promote environmentally friendly and resilient farming. Gender equality initiatives should be supported by policymakers to diversify and increase women's participation in green farming and rural entrepreneurship.

The findings highlight the importance of agricultural policies in shaping farmers' pro-environment and climate-friendly behaviour. AECS measures in the CAP encourage farmers to adopt green practices. Policymakers should focus on understanding the motives and factors that encourage farmers, particularly women-headed farms, to participate in AECS. To promote resilience and sustainability, farmers can be encouraged to participate in such programmes with tailored policy interventions and incentives.

The study also emphasises the importance of considering geographical and local contexts when studying AECS participation. Policymakers should consider local conditions and factors when creating AECS adoption policies. Education and training for rural women can boost their green farming participation, helping AECS initiatives succeed.

The study recommends monitoring and adjusting CAP policies. As farm size and type of farming activities affect AECS adoption and intensity, policymakers should periodically evaluate and adapt policies to address changing challenges and opportunities. This adaptive approach can improve the sustainability benefits of agricultural policies.

Although this research offers insightful information, it is important to recognise its limitations. The research is mainly focused on Slovenia, a nation located in CEE, and it is possible that the conclusions cannot be fully applied to other EU members. Extrapolating the results should be done with caution because different countries may have very different natural, agricultural structures, and factor endowment contexts, socioeconomic situations, and policy environments.

The BO decomposition model, while effective in labour economics, is applied to AECS in this study, and its suitability may be subject to scrutiny. Future research should take into account the effectiveness of the model in capturing the complex nature of AECS adoption and intensity, and alternative methodologies could be investigated for a more thorough understanding.

Even though FADN offers useful data, the outcomes could be impacted by biases or shifts in the agricultural environment over time. For a more thorough examination of trends and patterns, researchers and policymakers should consider longitudinal studies and be aware of the temporal limitations of the data.

In sum, the research adds to a contribution to the literature on gender-driven participation and intensity in AECS in the EU country. The policy implications and acknowledgement of limitations provide a foundation for future studies to broaden and improve methodologies, explore various geographical contexts, and contribute to the continuous effort of sustainable and gender-inclusive agricultural practices.

## Data Availability

The data that support the findings of this study are available from the Ministry of Agriculture, Forestry and Food of the Republic of Slovenia but restrictions apply to the availability of these data, which were used under license for the current study, and so are not publicly available. The datasets used and/or analysed during the current study are available from the corresponding author on reasonable request.
